# Augmentation of BMP Signaling in Cranial Neural Crest Cells Leads to Premature Cranial Sutures Fusion through Endochondral Ossification in Mice

**DOI:** 10.1002/jbm4.10716

**Published:** 2023-02-23

**Authors:** Hiroki Ueharu, Haichun Pan, Xia Liu, Mamoru Ishii, Jessica Pongetti, Anshul K. Kulkarni, Folasade E. Adegbenro, Jaden Wurn, Robert E. Maxson, Hongchen Sun, Yoshihiro Komatsu, Honghao Zhang, Jingwen Yang, Yuji Mishina

**Affiliations:** ^1^ Department of Biologic and Materials Sciences & Prosthodontics University Michigan School of Dentistry Ann Arbor Michigan USA; ^2^ Department of Oral Pathology Hospital of Stomatology, Jilin University Changchun China; ^3^ Department of Biochemistry & Molecular Medicine Keck School of Medicine of University of Southern California Los Angeles California USA; ^4^ Department of Orthodontics and Pediatric Dentistry University Michigan School of Dentistry Ann Arbor Michigan USA; ^5^ Department of Pediatrics University of Texas Health Science Center Houston Texas USA; ^6^ The State Key Laboratory Breeding Base of Basic Science of Stomatology (Hubei‐MOST) and Key Laboratory of Oral Biomedicine Ministry of Education School and Hospital of Stomatology, Wuhan University Wuhan China

**Keywords:** BMP SIGNALING, CELL FATE, CRANIOSYNOSTOSIS, P0‐Cre, Wnt1‐Cre

## Abstract

Craniosynostosis is a congenital anomaly characterized by the premature fusion of cranial sutures. Sutures are a critical connective tissue that regulates bone growth; their aberrant fusion results in abnormal shapes of the head and face. The molecular and cellular mechanisms have been investigated for a long time, but knowledge gaps remain between genetic mutations and mechanisms of pathogenesis for craniosynostosis. We previously demonstrated that the augmentation of bone morphogenetic protein (BMP) signaling through constitutively active BMP type 1A receptor (caBmpr1a) in neural crest cells (NCCs) caused the development of premature fusion of the anterior frontal suture, leading to craniosynostosis in mice. In this study, we demonstrated that ectopic cartilage forms in sutures prior to premature fusion in *caBmpr1a* mice. The ectopic cartilage is subsequently replaced by bone nodules leading to premature fusion with similar but unique fusion patterns between two neural crest‐specific transgenic Cre mouse lines, *P0‐Cre* and *Wnt1‐Cre* mice, which coincides with patterns of premature fusion in each line. Histologic and molecular analyses suggest that endochondral ossification in the affected sutures. Both in vitro and in vivo observations suggest a greater chondrogenic capacity and reduced osteogenic capability of neural crest progenitor cells in mutant lines. These results suggest that the augmentation of BMP signaling alters the cell fate of cranial NCCs toward a chondrogenic lineage to prompt endochondral ossification to prematurely fuse cranial sutures. By comparing *P0‐Cre;caBmpr1a* and *Wnt1‐Cre;caBmpr1a* mice at the stage of neural crest formation, we found more cell death of cranial NCCs in *P0‐Cre;caBmpr1a* than *Wnt1‐Cre;caBmpr1a* mice at the developing facial primordia. These findings may provide a platform for understanding why mutations of broadly expressed genes result in the premature fusion of limited sutures. © 2022 The Authors. *JBMR Plus* published by Wiley Periodicals LLC on behalf of American Society for Bone and Mineral Research.

## Graphical abstract

Craniosynostosis is characterized by premature fusion of cranial sutures resulting in abnormal skull shapes. Here, we showed that augmentation of BMP signaling alters cell fate of cranial neural crest cells towards chondrogenic lineage and that develops ectopic cartilage, which leads to premature fusion of cranial sutures through endochondral ossification.

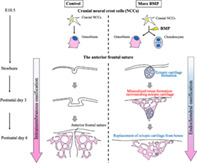

## Introduction

Cranial sutures are fibrous tissues connecting bones of the head and face and function as signaling centers to support the continuous growth of facial and calvarial bones.^(^ ^1^, ^2^, ^3^ ^)^ Many sutures eventually fuse during postnatal development in humans. When the sutures prematurely fuse as a result of genetic mutations or other biological reasons, especially in young ages, this causes a pathologic condition called craniosynostosis.^(^ ^2^, ^3^, ^4^ ^)^ Craniosynostosis leads to abnormal skull shape and increased intracranial pressure, potentially resulting in blindness, deafness, and some cases mental retardation. The incidence is ~1 in 2,500 births.^(^ ^2^ ^)^ Current treatment is limited to repetitive surgeries to remove the prematurely fused sutures; these repeated surgeries also have negative impacts on patients’ quality of life and impose a significant financial burden.^(^ ^5^, ^6^, ^7^ ^)^ Thus, understanding the molecular and cellular pathogenesis of craniosynostosis may contribute to the development of strategies for early identification, prevention, and treatment of these conditions.

Cranial neural crest cells (NCCs) are the progenitor population giving rise to the major components of the face and the anterior part of the head.^(^ ^8^, ^9^ ^)^ Cranial NCCs are multipotent cells that differentiate into osteoblasts, chondrocytes, adipose, and other ectoderm‐derived tissues.^(^ ^10^, ^11^ ^)^ Dysregulations of cranial NCCs, for example, aberrant migration, cell death, proliferation, differentiation, and cell fate specification, have been investigated as causative factors in craniofacial defects such as cleft palate and craniosynostosis.^(^ ^12^, ^13^, ^14^ ^)^ Cranial NCCs and suture development in mice are similar to those in humans. Therefore, mouse models are valuable tools in determining the etiology and potential therapeutic strategies to treat craniosynostosis.

Several signaling pathways are reported to regulate the mechanisms of cranial NCCs. We and others have reported that transgenic mice with gain‐of‐function mutations or loss‐of‐function mutations for bone morphogenetic protein (BMP) signaling in NCCs develop craniofacial defects.^(^ ^15^, ^16^, ^17^ ^)^ We generated transgenic mice expressing constitutively active BMP type 1A receptor (*caBmpr1a*) in a NCC‐specific manner using a *P0‐Cre* mouse line to demonstrate that *P0‐Cre;caBmpr1a* mice prematurely fused the anterior frontal (AF) suture, leading to a craniosynostosis.^(^ ^15^, ^18^, ^19^ ^)^ Interestingly, *P0‐Cre;caBmpr1a* mice developed premature fusion in the AF suture but other sutures such as the coronal and sagittal sutures remained patent.^(^ ^15^ ^)^ Despite broader expressions of the responsive genes in the craniofacial region, a fusion in limited sutures is frequently observed in patients with craniosynostosis and model mice for craniosynostosis.^(^ ^20^, ^21^ ^)^ As a result of the complexity of diseases, the underlying mechanisms remain poorly understood.

As the first step toward understanding a mechanism leading to a suture‐specific premature fusion by BMP signaling, we selected two neural crest‐specific Cre transgenic mice lines, *Wnt1‐Cre* and *P0‐Cre*, of which Cre expression is driven by *Wnt1* promoter and *Protein zero* (*P0*) promoter, respectively.^(^ ^8^, ^22^, ^23^, ^24^, ^25^ ^)^ They show similar, but not identical, Cre expression patterns of cranial NCCs at early embryonic stages.^(^ ^25^, ^26^, ^27^ ^)^ We previously showed that neural crest‐specific disruption of *Evc2*, a causative gene for Ellis–van Creveld syndrome, by *Wnt1‐Cre* mice or *P0‐Cre* mice, develops a different severity of craniofacial deformity due to their different recombination efficiency in the skull base.^(^ ^28^ ^)^ We thus expected that differential augmentation patterns of BMP signaling using two Cre transgenic lines might highlight a suture‐specific behavior of cranial NCCs with BMP signaling.

Endochondral ossification is one of two mechanisms for bone formation, which develops cartilage tissues first. Mesenchymal cells at an early embryonic stage form high‐density cell aggregations and start to express *Sox9*, a chondrogenic master gene, to initiate chondrogenesis. The resulting cartilage is gradually replaced by bone tissue.^(^ ^29^, ^30^ ^)^ Intramembranous ossification is another mechanism that plays a role for calvaria development. Mesenchymal cells in osteogenic fronts at cranial sutures differentiate into osteoblasts to form bones without the formation of cartilage. However, several reports demonstrated the potential involvement of endochondral ossification at the moment of suture fusion, both in physiologic and pathologic instances. Unlike humans, most sutures in mice stay patent throughout their life, except the posterior frontal (PF) suture, which normally fuses by postnatal day 15 (P15).^(^ ^31^ ^)^ Prior to the fusion of the PF suture under normal physiological condition, the formation of cartilage tissue is demonstrated, and the subsequent disappearance of the cartilage coincides with suture fusion.^(^ ^31^ ^)^ Similarly, the presence of cartilage in PF sutures in rats and metopic sutures in humans has been reported.^(^ ^32^, ^33^ ^)^ In pathological conditions, ectopic cartilage formation is found in the sagittal suture of *Axin2* mutant mice before its premature fusion, likely due to increased Wnt signaling activity.^(^ ^34^ ^)^ Interestingly, ectopic cartilage was also identified at the coronal suture in knock‐in mouse carrying a gain‐of‐function mutation of fibroblast growth factor receptor 2 (FGFR2) before premature suture fusion.^(^ ^35^ ^)^ These findings suggest that ectopic cartilage promotes endochondral ossification within the suture mesenchyme, resulting in premature fusion of cranial sutures in both rodents and humans.

In this study, we demonstrated that enhanced BMP signaling in NCCs leads to the development of ectopic cartilage in sutures that subsequently undergo premature fusion. We employed two NCC‐specific Cre lines, *P0‐Cre* and *Wnt1‐Cre*, and the resulting mice show similar but unique patterns of ectopic cartilage formation that coincides with their respective premature fusion patterns. These results suggest that endochondral ossification through ectopic cartilage formation is a mechanism that may lead to premature suture fusion. Despite identical Cre recombination patterns between *P0‐Cre* and *Wnt1‐Cre* at the perinatal stages, we found different levels of cell death in the medial nasal process (MNP) at E10.5, which is a putative origin of suture mesenchyme. These results imply that cranial NCCs may be primed by the augmentation of BMP signaling soon after their birth, which subsequently influences their behavior at perinatal stages for craniofacial development.

## Materials and Methods

### Mouse breeding

The mouse line carrying the Cre‐inducible constitutively activated *Bmpr1a* (*caBmpr1a*) transgene was described previously.^(^ ^15^ ^)^ *caBmpr1a* were bred with *Wnt1‐Cre* mice (B6.Cg‐Tg(Wnt1‐cre)11Rth Tg(Wnt1‐GAL4)11Rth/J, Jackson Lab, Stock No. 009107)^(^ ^22^ ^)^ or *P0‐Cre* mice (C57BL/6JTg(P0‐Cre)94Imeg (ID 148) provided by CARD, Kumamoto University, Japan)^(^ ^23^ ^)^ to obtain two distinct patterns of augmented BMP signaling in NCCs (henceforth *Wnt1‐Cre;caBmpr1a* and *P0‐Cre;caBmpr1a*). To visualize NCCs, *Wnt1‐Cre* and *P0‐Cre* mice were crossed with Cre reporter lines: Rosa26‐LacZ^(^ ^36^ ^)^ or tdTomato (B6.Cg‐Gt(ROSA)26Sortm14(CAG‐tdTomato)Hze/J, Jackson Lab, Stock No. 007914).^(^ ^37^ ^)^ All mice used were group housed in specific pathogen‐free conditions, fed a regular rodent diet, and kept in a healthy state. For each of the methods outlined, both male and female mice were utilized. All animals and embryos were allocated to experimental groups based on their genotypes, and there were no excluded animals and embryos. Investigators were blinded during allocation, animal handling, and endpoint measurements. All animal experiments were performed in accordance with the policies and federal laws associated with the judicious use of vertebrate animals as approved by the Institutional Animal Care and Use Committee (IACUC) at the University of Michigan (PRO00009613) and were conducted in accordance with ARRIVE guidelines.

### Micro–computed tomography

Mice were euthanized at postnatal day 17 (P17), and heads were harvested, debrided of extra tissues, and fixed with 10% formalin overnight. The heads were then embedded in 1% agarose, placed in a 19‐mm‐diameter tube, and scanned over their whole length using a micro–computed tomography (μCT) system (μCT40 Scanco Medical). Bone surface images were generated as described previously.^(^ ^15^, ^19^ ^)^

### Image segmentation and surface models

For quantification of the skull morphological differences, surface models were generated from CT scans through ITK‐SNAP (www.itksnap.org).^(^ ^38^ ^)^ Then anatomical landmarks were placed on the models according to Tables S1 and S2, which were also shown in Figure S1,^(^ ^39^, ^40^ ^)^ using 3D Slicer (www.slicer.org). The linear distances between landmarks were determined by the Q3DC module in 3D Slicer. There were no significant gender differences in either the control or the mutants, and the data from both genders are shown and compared between genotypes in Tables 1, S3, and S4.

**Table 1:** *P0‐Cre;caBmpr1a* and *Wnt1‐Cre;caBmpr1a* mutant mice demonstrate differential skull defects.

		Nasomaxillary complex						Calvaria					Cranial base				Viscerocranial heights			Mandibular analysis	
	Total skull length	Nasal bone length	Frontal bone length	Width of nasal bone at intersection with premaxillae	Zygomatic arch length	Erupted upper incisor length	Viscerocranial length II	Parietal length	Interparietal length	Width at zygomatic arch (anterior)	Width at zygomatic arch (posterior)	Width of temporal bone	Cranial Base length	Presphenoid length	Basiosphenoid length	Basiooccupital length	Viscerocranial height at ISS	Viscerocranial height at SOS	Viscerocranial height at Basion	Erupted lower incisor length	Width of mandible at condyle
Landmark (see Table S2)	43‐9	43‐44	44‐45	10‐11	47‐48	51‐52	45‐51	45‐46	46‐9	14‐15	18‐19	20‐21	43‐29	26‐27	27‐28	28‐29	45‐27	45‐28	45‐29	53‐55	41‐42
Description	A‐Pri	A‐N	N‐F	XN‐XN	ZMx‐ZT	MxAl‐MxC	F‐MxAl	F‐Pr	Pr‐Pri	ZMx‐ZMx	ZT‐ZT	Tp‐Tp	A‐Ba	Sp‐Iss	Iss‐Sos	Sos‐Ba	F‐Iss	F‐Sos	F‐Ba		
Control average (*n* = 15)	18.802467	5.922000	6.253800	2.462333	3.711200	1.141800	11.999467	4.206400	3.436533	8.824200	10.432800	9.868333	16.801400	2.520200	2.911867	3.052533	5.739000	6.578267	8.625267	1.874533	9.246800
Control SD	0.295319	0.118179	0.154106	0.076442	0.151978	0.202060	0.183480	0.230933	0.247256	0.150652	0.207980	0.120044	0.194174	0.077444	0.097175	0.045649	0.129511	0.150184	0.141400	0.151644	0.231719
P0‐mut Ave (*n* = 10)	15.611800	4.266300	4.986400	2.088400	3.558100	1.286300	10.068800	4.273500	3.396600	9.049600	10.215400	9.262400	13.487200	2.203900	2.341400	2.906700	5.584500	6.507300	8.422100	1.677900	9.3544
P0‐mut SD	2.110937	1.198884	0.986993	0.385340	0.328989	0.300739	1.576280	0.359444	0.242757	0.981250	0.230200	0.862823	2.074656	0.416877	0.326408	0.222166	0.132983	0.071052	0.149560	0.155097	0.336505968
T Test cont vs. P0	0.000006	0.000019	0.000055	0.001215	0.128005	0.162759	0.000088	0.573909	0.693989	0.386144	0.022091	0.012508	0.000002	0.008178	0.000002	0.020443	0.008232	0.178701	0.002229	0.004504	0.352055807
Control average (*n* = 4)	17.722500	4.585500	6.494750	1.672000	4.022000	1.135250	11.812000	4.174250	3.479750	8.641500	10.310000	9.428500	15.740250	2.521000	2.639000	3.092750	5.743500	6.590000	8.625500	1.928000	9.432250
Control SD	0.301333	0.399934	0.193014	0.214375	0.362724	0.174748	0.143826	0.388229	0.101385	0.118498	0.193360	0.308412	0.334321	0.073652	0.468897	0.366078	0.055836	0.186312	0.208628	0.121147	0.137723
Wnt‐mut Ave (*n* = 5)	15.304600	3.492400	5.709800	1.677600	3.980600	1.410000	11.211400	4.383400	3.342600	8.469200	10.521600	9.622000	13.115600	2.600600	2.103000	2.845800	6.032800	6.758600	8.514000	1.834400	9.521400
Wnt‐mut SD	0.799786	0.749974	0.426760	0.186880	0.342269	0.277489	0.531976	0.387872	0.140514	0.248400	0.459869	0.291404	0.662011	0.210919	0.301208	0.146691	0.506133	0.539445	0.467605	0.293347	0.420669
T Test cont vs. Wnt	0.000760	0.034942	0.011801	0.967731	0.865477	0.130211	0.066835	0.448100	0.146628	0.246613	0.422079	0.366536	0.000183	0.499283	0.074901	0.205941	0.298838	0.573420	0.674152	0.572167	0.699625

*Note*: Summary of 21 measurements are shown in millimeters. Data for each sample are shown in Table S3 (*P0‐Cre;caBmpr1a* mice) and Table S4 (*Wint1‐Cre;caBmpr1a* mice). Significant differences with control littermates are highlighted (yellow, common for *P0‐Cre* and *Wnt1‐Cre*; green, only for *P0‐Cre*). Student's *t* test was used for statistical analysis

### Histology, cartilage staining, and immunohistochemistry

Embryos and postnatal heads at newborn (NB), postnatal day 10 (P10), and P17 were harvested and fixed with 4% paraformaldehyde (PFA) overnight at 4°C. Tissues were then immersed with 30% sucrose in PBS until the tissues sank. The tissues were embedded in optimal cutting temperature compound (Thermo Fisher Scientific), and 10‐μm cryosections were prepared by cryostat (Leica CM1850). Hematoxylin and eosin (H&E) staining, Alcian blue staining, and immunohistochemistry for SOX9, pSMAD1/5/9, and TUNEL measurement were done as described previously.^(^ ^15^, ^18^, ^19^, ^41^ ^)^ The primary antibodies used are as follows: rabbit antibody against human SOX9 (1:500 dilution, Millipore, AB5535), rabbit antibody against human phospho‐SMAD 1 (Ser463/465)/Smad5 (Ser463/465)/ Smad9 (Ser465/467) (1:200 dilution, Cell Signaling, 13820 S), rabbit antibody against human COL1 (1:200 dilution, Abcam, ab34710), mouse antibody against chick COL2 (1:200 dilution, Thermo Fisher Scientific, MA5‐12789), rabbit antibody against rat COL10 (1:100 dilution, Cosmo Bio., LSL‐LB‐0092), and rabbit antibody against mouse CD31 (1:100 dilution, Cell Signaling, 77699 S).

### Isolation of cranial neural crest stem cells and cells of nasal process at E11.5

We followed a previously published method.^(^ ^11^ ^)^ In brief, we isolated the cranial region of *P0‐Cre;caBmpr1a;mTmG* embryos at E8.5, and dissociated cells were cultured on a Matrigel‐coated dish using media containing 10^3^ units/mL of leukemia inhibitory factor and 25 ng/mL of FGF2. At passage 3, GFP‐positive cells were isolated using fluorescence‐activated cell sorting (FACS) and cultured further. Clones that were able to be cultured beyond passage 10 were assessed for expression of stem cell markers using quantitative RT‐PCR primers listed in Table S5. Their multipotency was assessed using media to prompt osteogenesis and pellet culture for chondrogenesis.^(^ ^11^ ^)^ The efficiency of establishing cranial neural crest stem cells (NCSCs) is summarized in Table S6. To isolate and differentiate cells of the nasal process (NP cells), we followed a previously published method.^(^ ^11^, ^41^ ^)^ Differentiation capacities were evaluated by alkaline phosphatase (ALP) staining, Alcian blue staining, and quantitative RT‐PCR for differentiation marker genes.^(^ ^11^, ^42^ ^)^

### Statistical analyses

Statistical analyses were performed using GraphPad Prism version 9.0 software. For each dataset, Student's *t* test for the comparison of two groups of mice and one‐way ANOVA with Tukey's test for multiple comparisons between groups of mice were performed. The *p* value is indicated by asterisks: * *p* < 0.05 and ** *p* < 0.01.

## Results

### Augmentation of BMP signaling in NCCs showed common and specific premature suture fusion patterns by crossing with *Wnt1‐Cre* and *P0‐Cre* mice

**Fig. 1:**
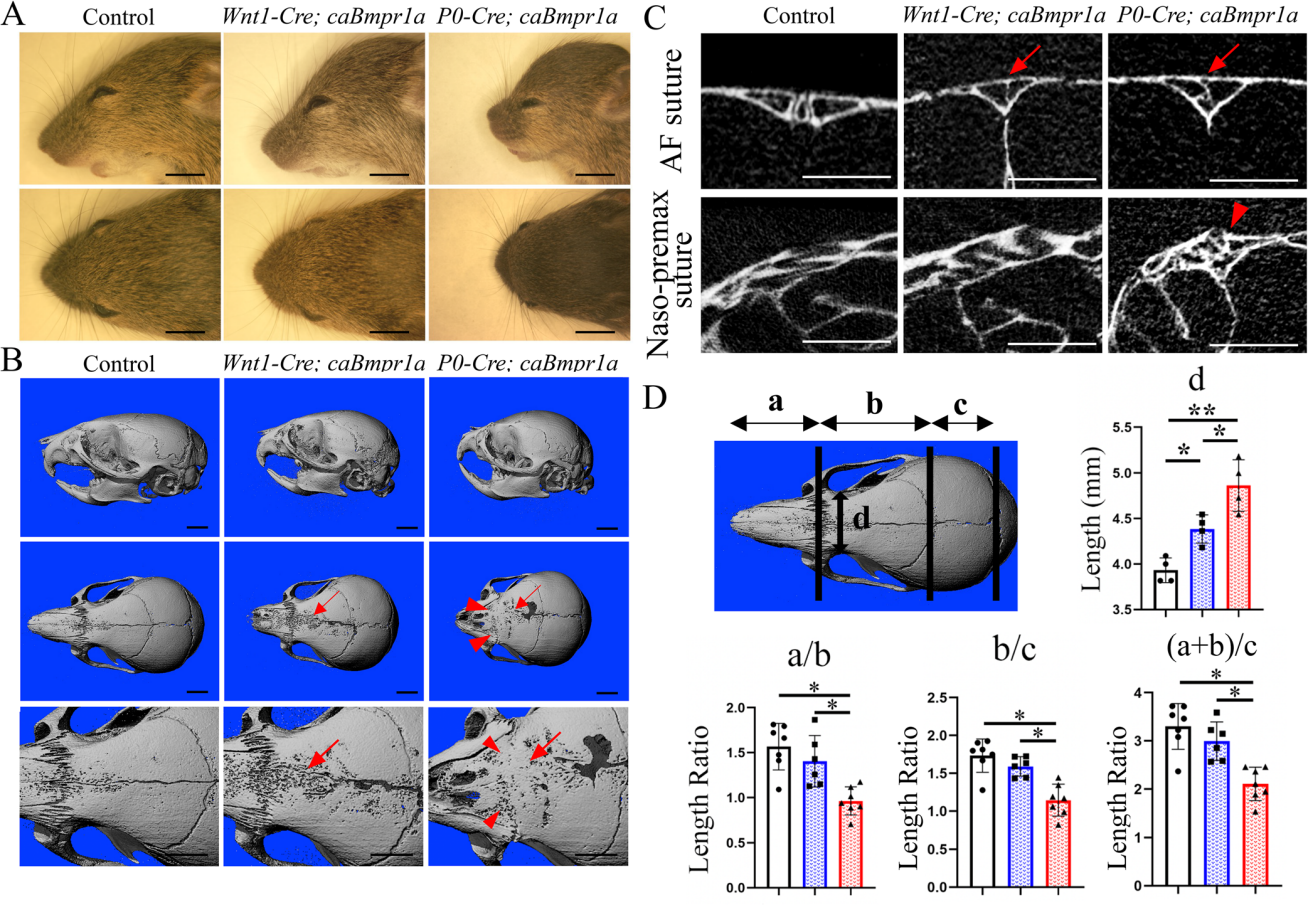
Skull deformities of *Wnt1‐Cre;caBmpr1a* and *P0‐Cre;caBmpr1a* mice. (*A*) Representative lateral and top views of head of *Wnt1‐Cre;caBmpr1a* and *P0‐Cre;caBmpr1a* mice at postnatal day 17 (P17) (*n* = 6). *Wnt1‐Cre;caBmpr1a* and *P0‐Cre;caBmpr1a* mice display short/broad snouts and hypertelorism. (*B*, *C*) Micro‐computed tomography (μCT) image at P17 showed suture fusion in *Wnt1‐Cre;caBmpr1a* (*n* = 11) and *P0‐Cre;caBmpr1a* (*n* = 6) mice. Representative lateral view, top view, and enlarged top view are shown (B). Two‐dimensional sections of μCT image at AF suture and naso‐premaxillary (naso‐premax) suture (*C*). Sutures that caused premature fusion in mutant mice are indicated by red arrows (AF suture) and red arrowheads (naso‐max suture), respectively. (*D*) Length of the nasal bone (*a*), the frontal bone (*b*), the parietal bone (*c*), and between eyes (*d*) is measured using μCT image. Relative length of the nasal bone divided by length of the frontal bone (*a*/*b*), length of the frontal bone divided by length of the parietal bone (*b*/*c*), and length of the nasal and frontal bone divided by length of the parietal bone ((*a* + *b*)/*c*) are quantified. White bars, control mice; blue bars, *Wnt1‐Cre;caBmpr1a* mice; red bars, *P0‐Cre;caBmpr1a* mice. Student's *t* test or one‐way ANOVA with Tukey's test were used for the statistical analysis. *p < 0.05, **p < 0.01. Scale bars: 5 mm (*A*), 2 mm (*B*), 1 mm (*C*).

To investigate the function of BMP signaling in cranial NCC development, we crossed *caBmpr1a* with *P0‐Cre* mice (hereafter, *P0‐Cre;caBmpr1a* mice) and *Wnt1‐Cre* mice (hereafter, *Wnt1‐Cre;caBmpr1a* mice). Both *Wnt1‐Cre;caBmpr1a* mice and *P0‐Cre;caBmpr1a* mice developed short snouts, round faces, and hypertelorism at P17 (Fig. 1*A*). μCT images showed premature fusion of the AF suture in both *Wnt1‐Cre;caBmpr1a* and *P0‐Cre;caBmpr1a* mice (Fig. 1*B*,*C*, red arrows). Notably, the naso‐premaxillary suture in *P0‐Cre;caBmpr1a* was also prematurely fused, but that in *Wnt1‐Cre;caBmpr1a* mice was not (Fig. 1*B*,*C*, red arrowheads). μCT images revealed that a ratio of the length between nasal bone, frontal bone, and parietal bone was significantly decreased in both *P0‐Cre;caBmpr1a* and *Wnt1‐Cre;caBmpr1a* mice compared with controls, but more significant in *P0‐Cre;caBmpr1a* mice (Fig. 1*D*). On the other hand, the distance between eyes was significantly greater in both *P0‐Cre;caBmpr1a* and *Wnt1‐Cre;caBmpr1a* mice compared with controls; furthermore, the length was significantly longer in *P0‐Cre;caBmpr1a* than *Wnt1‐Cre;cBmpr1a* mice (Fig. 1*D*).

We then carried out a more detailed analysis of the skull shape. We placed landmarks on skull models according to Tables S1 and S2 to determine multiple linear distances. In the comparison of *P0‐Cre;caBmpr1a* mice with littermate controls, we observed significant differences in 14 out of 21 measurements, including skull length, nasal bone length, frontal bone length, width of nasal bone at the intersection with premaxillae, viscerocranial length, width at zygomatic arch, width of temporal bone, cranial base length, presphenoid length, basisphenoid length, basioccipital length, viscerocranial height at intersphenoid synchondrosis (ISS), viscerocranial height at bason, and erupted lower incisor length (Tables 1 and S3). In contrast, only four measurements, skull length, nasal bone length, frontal bone length, and cranial base length, showed differences in *Wnt1‐Cre;caBmpr1a* mice, and two other measurements, viscerocranial length and basisphenoid length, showed a tendency toward reduction (Table 1 and S2). These differences highlight similar but not identical skull defects in *P0‐Cre;caBmpr1a* and *Wnt1‐Cre;caBmpr1a* mice.

**Fig. 2:**
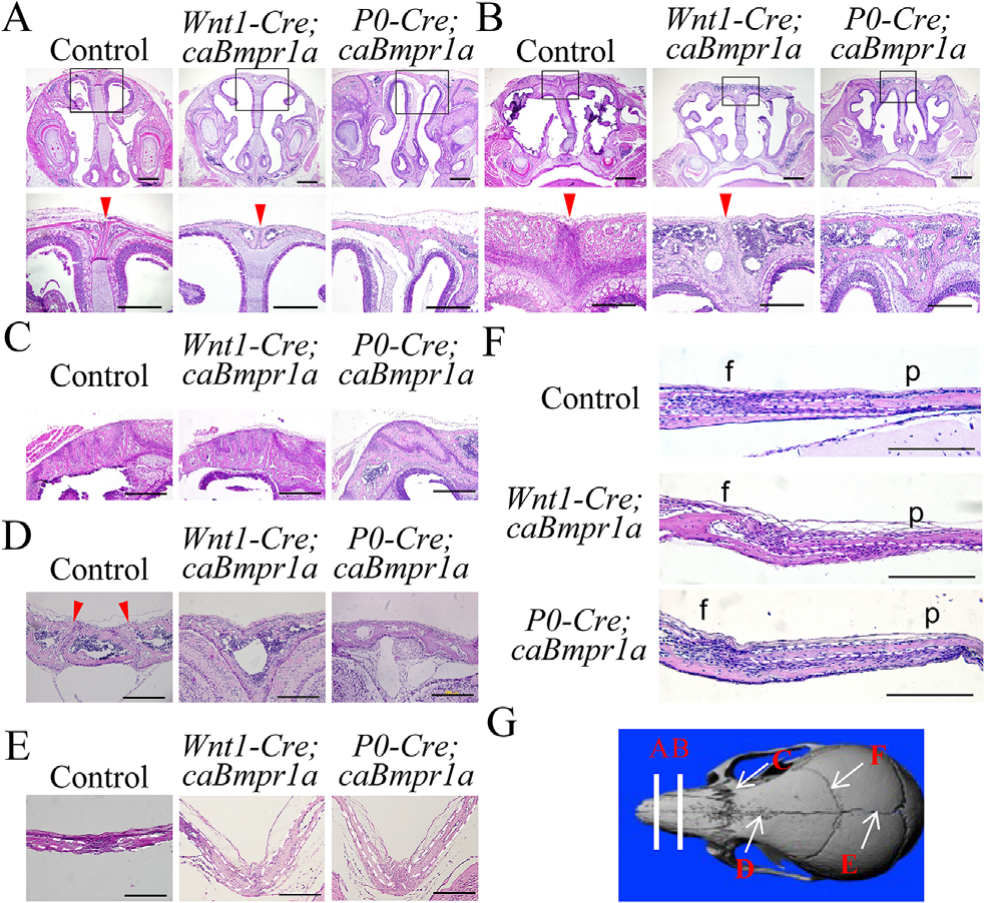
Histological observation of respective sutures in control, *Wnt1‐Cre;caBmpr1a* mice, and *P0‐Cre;caBmpr1a* mice. (*A*–*F*) Hematoxylin and eosin staining for nasal‐frontal suture (*A*), nasal‐posterior suture (*B*), naso‐premaxillary suture (*C*), anterior frontal suture (*D*), sagittal suture (*E*), and coronal suture (*F*) of control mice (*n* = 6), *Wnt1‐Cre;caBmpr1a* mice (*n* = 6), and *P0‐Cre;caBmpr1a* mice (*n* = 6), respectively. Frontal bone (f) and posterior bone (p) of coronal suture. (*G*) Schematic image of mouse skull indicating sutures. Arrowheads indicate patent sutures. Scale bars: 1 mm (*A*–*F*), 500 mm (*A*, *B*, boxed area), and 200 μm (*C*–*F*).

Histological assessments at P17 demonstrated that the nasofrontal and nasoposterior suture (Fig. 2*A*,*B*), the naso‐premaxillary suture (Fig. 2*C*), and the AF suture (Fig. 2*D*) were deformed and prematurely fused in *P0‐Cre;caBmpr1a* mice, while in *Wnt1‐Cre;caBmpr1a* mice only the AF suture showed premature fusion (Fig. 2*A*–*D*). The sagittal suture (Fig. 2*E*) and the coronal suture (Fig. 2*F*) were not fused in either mutant line as expected from the μCT images. These results suggest that common and specific sutures prematurely fused in *Wnt1‐Cre;caBmpr1a* and *P0‐Cre;caBmpr1a* mice.

### Ectopic cartilage formed in suture where prematurely fused

We recently reported that constitutively activated ACVR1, another BMP receptor type1, in NCCs (henceforth *caAcvr1;P0‐Cre* mice) developed ectopic cartilage in the developing face.^(^ ^41^ ^)^ It has been reported that the PF suture fuses via endochondral ossification under normal physiological conditions.^(^ ^31^ ^)^ Based on these reports, we hypothesized that enhanced BMP signaling in NCCs altered cell fate to develop ectopic cartilages within suture mesenchyme, which prompts endochondral ossification, resulting in premature suture fusion.

**Fig. 3:**
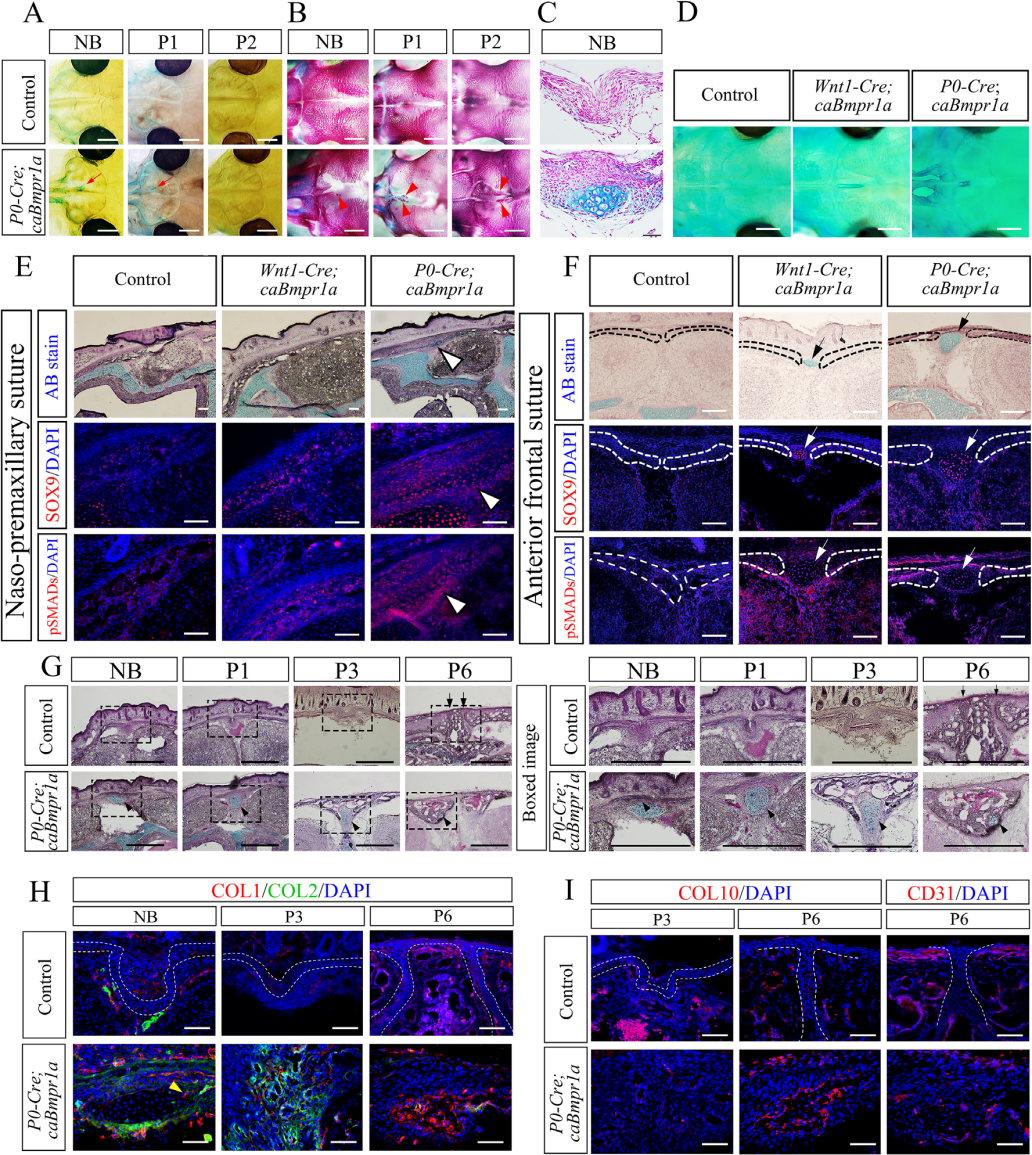
Spatial correlations of ectopic cartilage and premature fusion of cranial sutures in *Wnt1‐Cre;caBmpr1a* and *P0‐Cre;caBmpr1a* mice. (*A*) Ectopic cartilage in head stained with Alcian blue solution at newborn (NB), postnatal day 1 (P1), and P2 (*n* = 6). Ectopic cartilage in anterior frontal (AF) suture is indicated by red arrows. (*B*) Calvaria formation of control mice and *P0‐Cre;caBmpr1a* mice stained with Alizarin red solution at NB, P1, and P2. Ectopic bone nodules in the AF suture are indicated by red arrowheads. (*C*) Ectopic cartilage in AF suture of *P0‐Cre;caBmpr1a* mice at NB was histologically detected with Alcian blue solution. (*D*) Whole cartilage staining with Alcian blue of control, *Wnt1‐Cre;caBmpr1a*, and *P0‐Cre;caBmpr1a* mice at NB are shown. (*E*, *F*) Ectopic cartilage in naso‐premaxillary suture (*E*) and AF suture (*F*) were histologically stained with Alcian blue, SOX9, and pSMAD 1/5/9 (*n* = 6). The ectopic cartilage in the naso‐premaxillary suture is indicated by white arrowheads, and the ectopic cartilage in the AF suture is indicated by white arrows. Osteogenic front of both the naso‐premaxillary suture and the AF suture are indicated by dotted lines. (*G*) Histological assessments for presence of ectopic bone formation in relation to positions of ectopic cartilage by Alcian blue staining followed by hematoxylin staining. Black arrows indicate patent AF suture. Black arrowheads indicate ectopic cartilage. Boxed area in (*G*) is enlarged. (*H*, *I*) Double immunohistochemistry for COL1 (red) and COL2 (green) at NB, P3, and P6 (*H*), and immunohistochemistry for COL10 or CD31 (*I*) are shown. A yellow arrowhead in (*H* **)** indicates a COL1‐positive cell in ectopic cartilage at NB. Nuclei were stained with DAPI. Dotted lines indicate AF suture in control mice. Scale bars: 1 mm (*A*, *B*, *D*), 100 μm (*C*, *E*, *F*, *H*, *I*), 500 μm (*G*).

We next identified cartilage tissues in the sutures after the birth of *P0‐Cre;caBmpr1a* mice by whole mount cartilage staining with Alcian blue. Ectopic cartilages formed at positions of the AF suture and the naso‐premaxillary suture of *P0‐Cre;caBmpr1a* mice at NB and postnatal day 1 (P1), and the ectopic cartilage disappeared at P2 (Fig. 3*A* arrows). A small portion of bone nodule concomitantly formed at the AF suture at the NB stage, where the ectopic cartilage formed; subsequently, the size of bone nodules gradually increased in association with the disappearing ectopic cartilage (Fig. 3*B*, arrowheads). These results suggest that the ectopic cartilage was replaced by bone nodules. Histological observation confirmed Alcian blue‐stained cartilage‐like tissues were present in the AF suture of *P0‐Cre;caBmpr1a* mice (Fig. 3*C*). Interestingly, in *Wnt1‐Cre;caBmpr1a* mice, we identified ectopic cartilage only in the AF sutures, but not in the naso‐premaxillary suture at the NB stage (Fig. 3*D*).

We further histologically assessed fusion patterns of sutures in control, *Wnt1‐Cre;caBmpr1a*, and *P0‐Cre;caBmpr1a* mice. The naso‐premaxillary suture, which prematurely fused only in *P0‐Cre;caBmpr1a mice*, developed a small portion of ectopic cartilage tissue at the NB stage of *P0‐Cre;caBmpr1a* mice, but not in control and *Wnt1‐Cre;caBmpr1a* mice (Fig. 3*E*, arrowheads). There were also ectopic SOX9‐positive chondrogenic progenitor cells that are positive for phospho‐SMAD1/5/9 in the naso‐premaxillary suture (Fig. 3*E*, arrowheads). On the other hand, the AF suture, which prematurely fused in both *Wnt1‐Cre;caBmpr1a* and *P0‐Cre;caBmpr1a* mice, formed ectopic cartilage with cells positive for SOX9 and phospho‐SMAD1/5/9 (Fig. 3*F*, arrows). The AF suture in control mice did not develop ectopic cartilage.

Next, we examined whether the ectopic cartilage contributed to ectopic bone formation through endochondral ossification. We histologically assessed the ectopic cartilage in the AF suture in control and *P0‐Cre;caBmpr1a* mice at NB, P1, P3, and P6 (Fig. 3*G*). The ectopic cartilage was enclosed in ectopic bone tissue in *P0‐Cre;caBmpr1a* mice at P3; then the ectopic cartilage became smaller at P6 (Fig. 3 *G*, black allow heads). Endochondral ossification progresses by chondrocyte differentiation into matured hypertrophic chondrocytes and invasion of osteoblasts and blood vessels into the cartilage template. At the NB stage, a few COL2‐positive chondrocytes appeared at the ectopic cartilage. We also observed a small number of COL1‐positive cells in the ectopic cartilage at NB (Fig. 3*H*, yellow arrowhead). The numbers of COL2‐positive chondrocytes and COL1‐positive cells were higher at P3 than the NB stage. The number of COL2‐positive chondrocytes became lower at P6, while the number of COL1‐positive osteoblasts became higher (Fig. 3*H*). We further detected COL10‐positive cells, a marker for hypertrophic chondrocytes, and CD31‐positive cells, a marker for vascular endothelial cells, at P6 (Fig. 3*I*). Taken together, these data strongly suggest that ectopic cartilage is formed only in sutures that cause premature suture fusion and contribute to premature suture fusion by prompting endochondral ossification.

### Cranial NCSCs with enhanced BMP signaling showed higher capacity for chondrogenic differentiation

**Fig. 4:**
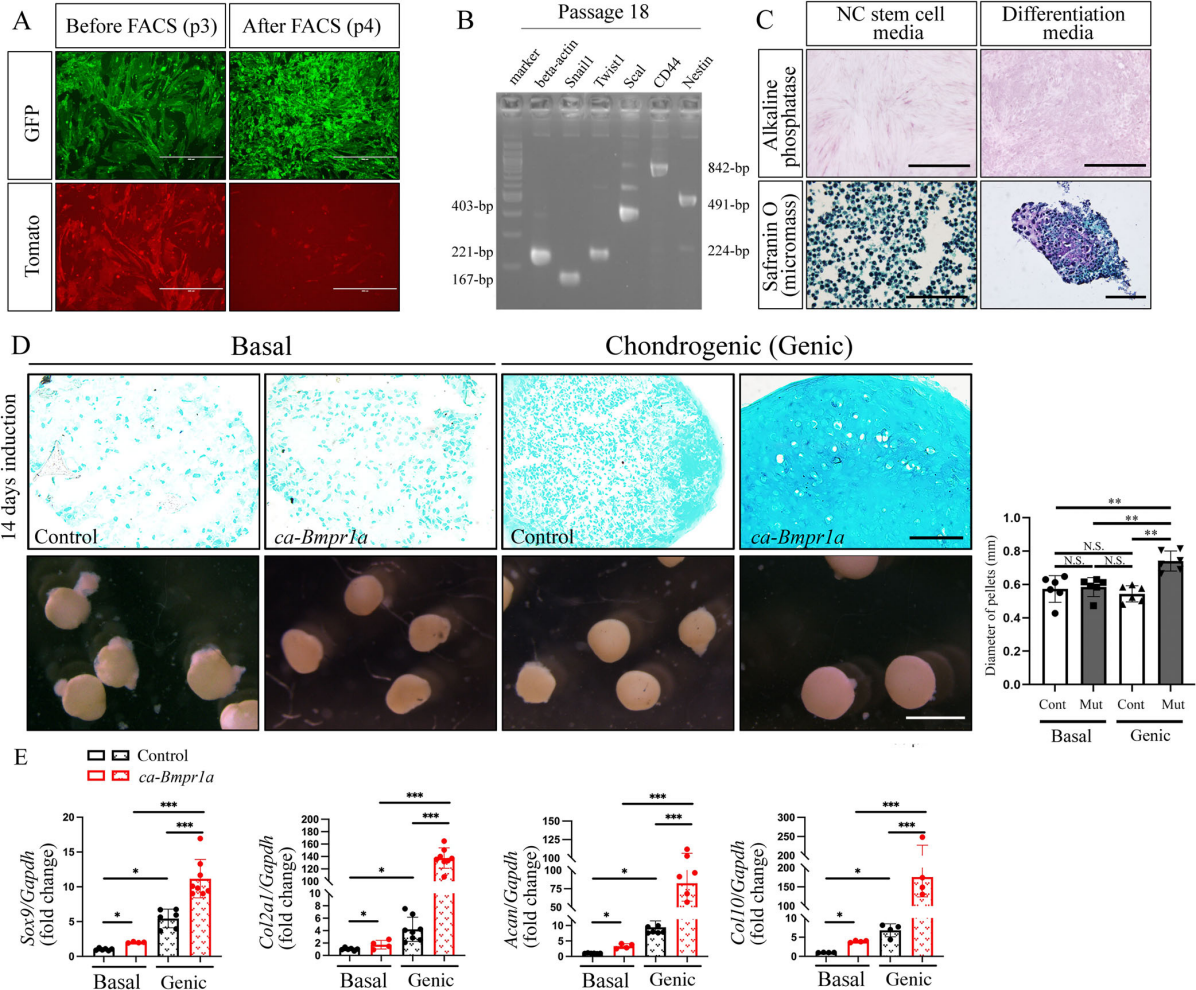
Cranial neural crest stem cells (NCSCs) with enhanced BMP signaling showed higher capacity for chondrogenic differentiation. (*A*) Cells isolated from control or *P0‐Cre;ca‐Bmpr1a* mutant embryos labeled with *R26R* ^*mTmG*^ reporter were visualized by fluorescent microscopy before and after FACS. (*B*) RT‐PCR results of neural crest and stem cell markers in NCSCs at passage 18 (*n* = 3). (*C*) Alkaline phosphatase staining and Safranin O staining of cranial NCSCs with or without osteogenic or chondrogenic induction, respectively (*n* = 3). (*D*, *E*) Chondrogenic induction of control and *P0‐Cre;caBmpr1a* cranial NCSCs after 14 days of culture with or without chondrogenic induction. Alcian blue staining and representative pellets are shown (*D*). The diameter of the pellets was measured. Relative gene expression of chondrogenic markers of control and *P0‐Cre;ca‐Bmpr1a* cranial NCSCs were quantified by quantitative RT‐PCR (*E*) (*n* = 6). Basal, cells cultured with basal medium; chondrogenic (Genic), cells cultured with chondrogenic medium. One‐way ANOVA with Tukey's test was used for statistical analysis. *p < 0.05, **p < 0.01, ***p < 0.001. Scale bars: 400 μm (*A*), 100 μm (*C*), 50 μm (*D*, sections), 1 mm (*D*, pellets).

Based on the ectopic cartilage formation in the sutures of mutant mice, we hypothesized that cranial NCCs with enhanced BMP signaling would show greater chondrogenic potential. To examine the chondrogenic differentiation potential of cranial NCCs, we isolated cranial NCCs from E8.5 mouse embryos to establish NCSC lines according to a previously published protocol.^(^ ^11^ ^)^ The cells were isolated from either control or *P0‐Cre;caBmpr1a* embryos labeled with an *R26R* ^*mTmG*^ reporter and GFP‐positive cells were purified at passage 3 using FACS (Fig. 4*A*). We found that the cell lines established expressed neural crest markers, *Snail1* and *Twist1*, and progenitor cell markers, *Nestin*, *CD44*, and *Sca‐1*, at 18 passages (Fig. 4*B*) by RT‐PCR analysis listed in Table S5. The cells were capable of differentiating into osteoblasts and chondrocytes, assessed by ALP staining and safranin O staining (Fig. 4*C*). We found that cranial NCCs from about 40% of embryos could grow over 10 passages regardless of their genotypes (Table S6). We further used 3D chondrogenic pellet cultures to examine the chondrogenic differentiation potential of NCSC lines from control and *P0‐Cre;caBmpr1a* embryos. After 14 days of chondrogenic induction, NCSCs from *P0‐Cre;caBmpr1a* embryos demonstrated more cartilage matrix deposition and larger pellet size than controls by Alcian blue staining (Fig. 4*D*) and higher expression levels of chondrogenic markers *Sox9*, *Col2a1*, *Acan*, and *Col10* by quantitative RT‐PCR (Fig. 4*E*). These results from NCSCs strongly suggest that cranial NCCs in *P0‐Cre;caBmpr1a* mice have a higher capacity for chondrogenic differentiation.

**Fig. 5:**
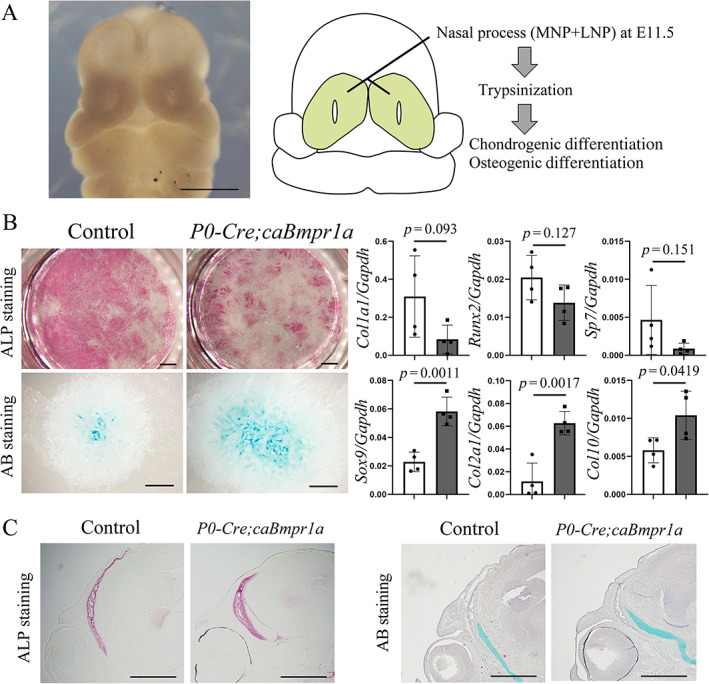
Cranial NCCs with enhanced BMP signaling showed a lower capacity for osteogenic differentiation. (*A*) Representative embryos at E11.5. Nasal process (NP) cells (green area) at E11.5 were isolated then induced chondrogenic and osteogenic differentiation. Capability of osteogenic differentiation was evaluated by alkaline phosphatase (ALP) staining and quantitative RT‐PCR for osteogenic marker genes. Capability of chondrogenic differentiation was evaluated by Alcian blue (AB) staining and quantitative RT‐PCR for chondrogenic marker genes. (*C*) The calvarial formation at E15.5 was assessed by ALP staining and AB staining. Student's *t* test was used for statistical analysis. Each *p* value is shown on the data. Scale bars: 1 mm (*A*, *B*), 500 μm (*C*).

It is well known that BMP signaling promotes osteogenic differentiation. Thus, we further examined whether BMP signaling affected the differentiation capability of osteogenic differentiation. We isolated the cells of nasal process (NP) cells at E11.5 then successively induced osteogenic and chondrogenic differentiation (Fig. 5*A*). The differentiation was assessed by ALP staining, Alcian blue staining, and quantitative RT‐PCR for osteogenic and chondrogenic marker genes. Chondrogenic differentiation was higher in *P0‐Cre;caBmpr1a* mice, as expected, and there was a lower osteogenic differentiation tendency in *P0‐Cre;caBmpr1a* mice in vitro (Fig. 5*B*). Frontal bones were hypomorphic in *P0‐Cre;caBmpr1a* mice at E15.5 (Fig. 5*C*, left). Taken together with a wider gap between the frontal bones at NB‐P2 (Fig. 3*B*), these results suggest that cranial NCCs in *P0‐Cre;caBmpr1a* mice have a lower capacity for osteogenic differentiation. Of note, cartilage formation in *P0‐Cre;caBmpr1a* mice was comparable to controls at E15.5 (Fig. 5*C*, right), suggesting that the impact of augmented BMP signaling in cell fate specification in suture progenitor cells becomes visible in later stages.

### Cranial NCCs marked by *Wnt1‐Cre* and *P0‐Cre* mice behave differently during early embryonic stage

**Fig. 6:**
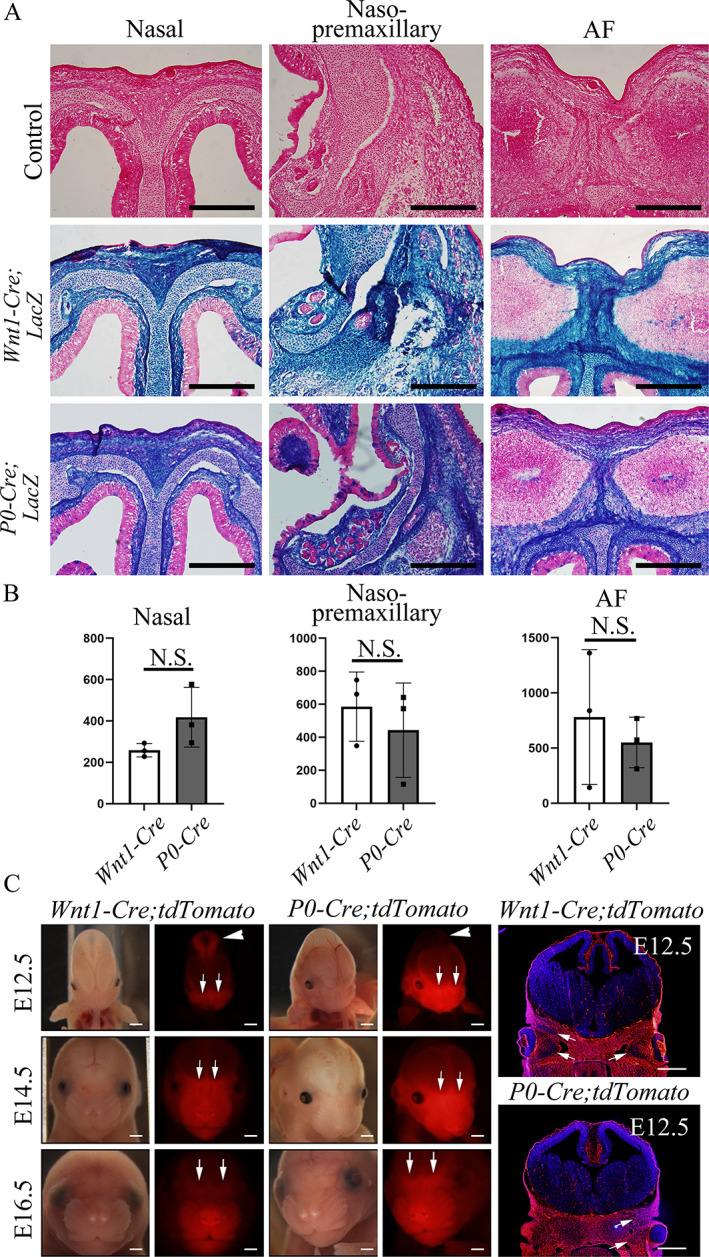
The distribution patterns of *Wnt1‐Cre* expressing and *P0‐Cre* expressing NCCs. *Wnt1‐Cre* and *P0‐Cre* mice were crossed with *tdTomato* and *LacZ* reporter mice to visualize NCCs, respectively. (*A*) NCCs derivatives at E16.5 were histologically revealed by LacZ staining (*n* = 6). Suture mesenchyme at nasal suture (nasal, left column), the premaxillary suture (premaxillary, center column), and anterior frontal (AF) suture (right column) are shown, respectively. (*B*) Quantification of LacZ‐negative area in suture mesenchyme. (*C*) NCCs at E12.5, E14.5, and E16.5 were visualized with *tdTomato* (*n* = 6). Representative sections of tdTomato staining in both *P0‐Cre* and *Wnt1‐Cre* mice at E12.5 are shown. Arrows indicate place showing higher tdTomato fluorescence in *P0‐Cre* mice than *Wnt1‐Cre* mice. Arrowheads indicate region where tdTomato signal is found in *Wnt1‐Cre* line but not in *P0‐Cre* line. Student's *t* test was used for statistical analysis. N.S., no significance. Scale bars: 500 μm (*A*, *C*).

Augmentation of BMP signaling in NCCs at an early embryonic stage alters cell fate toward a chondrogenic fate that develops ectopic cartilage. However, there is still a knowledge gap as to why the naso‐premaxillary suture prematurely fuses in *P0‐Cre;caBmpr1a* mice but not in *Wnt1‐Cre;caBmpr1a* mice, even though both Cre lines target NCCs. We then hypothesized that Cre‐mediated recombination patterns at early embryonic stages between *Wnt1‐Cre* and *P0‐Cre* mice are similar but not identical, which results in region‐specific premature suture fusions between two lines. To examine the differences in recombination patterns in neural crest (NC) derivatives between *Wnt1‐Cre* and *P0‐Cre* mice, we used *R26‐LacZ* mice to observe Cre recombination patterns by LacZ staining (hereafter, *Wnt1‐Cre;LacZ*, and *P0‐Cre;LacZ*). At E16.5, there were no overt differences in LacZ activities within the suture mesenchyme at the nasal suture, the naso‐premaxillary suture, and the AF suture (Fig. 6*A*,*B*). To detect small differences, we next employed tdTomato Cre‐reporter mice (*P0‐Cre;tdTomato* and *Wnt1‐Cre;tdTomato* mice) to observe the spatial distribution of red fluorescence at early embryonic stages (Fig. 6*C*). At E12.5, tdTomato fluorescence localized in the face in *Wnt1‐Cre* and *P0‐Cre* mice. *P0‐Cre* mice at E12.5 showed more intense tdTomato fluorescence than *Wnt1‐Cre* mice in the area that includes the presumptive naso‐premaxillary suture (Fig. 6*C*, arrows). Histological observation at E12.5 confirmed this notion (Fig. 6*C*, right). These small differences persisted at the three stages we observed (Fig. 6*C*). *Wnt1‐Cre* mice at 12.5 had tdTomato fluorescence at the hindbrain, which is known to be non‐NC‐derived (Fig. 6*C*, arrowheads).^(^ ^25^, ^43^, ^44^ ^)^ We and others have reported unique and overlapping patterns of *P0‐Cre* expressing and *Wnt1‐Cre* expressing cells in cranial NCCs at early embryonic stages when they start to emerge.^(^ ^25^ ^)^ Our result may suggest that cranial NCCs labeled by *P0‐Cre* are more involved in developing sutures in the anterior part of the face than those labeled by *Wnt1‐Cre* in a temporal‐specific manner before E16.5, which leads to the premature suture fusion of naso‐premaxillary sutures only in P0‐Cre mice.

**Fig. 7:**
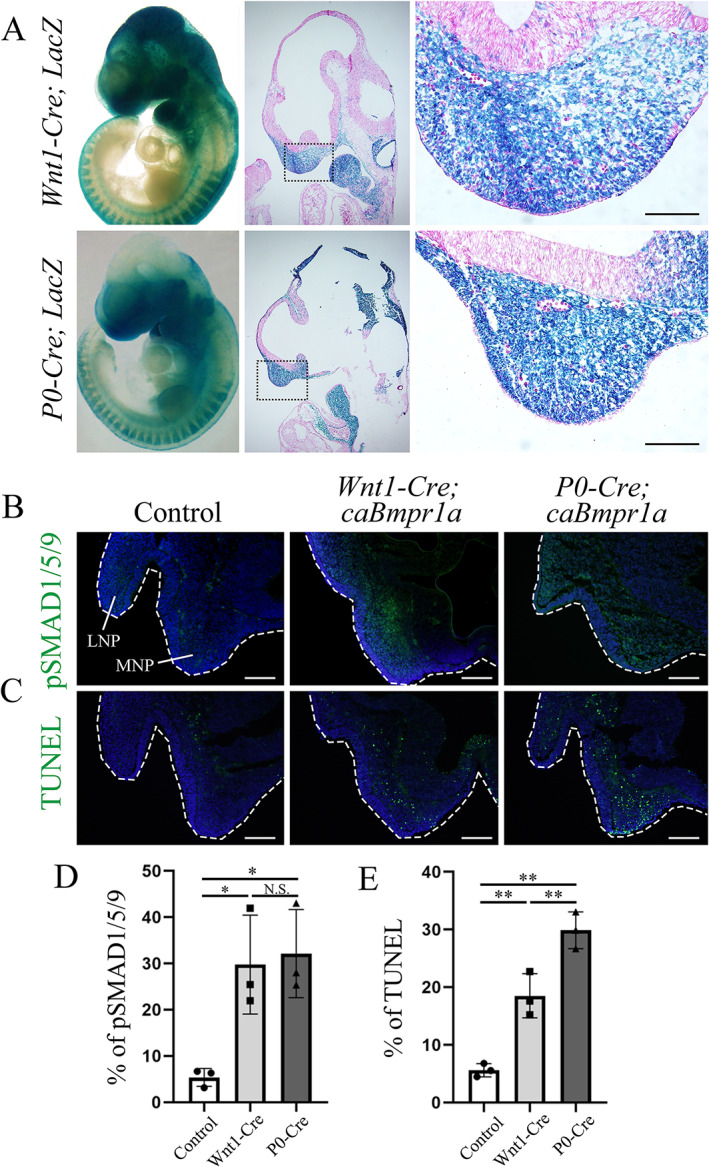
Effect of augmented BMP signaling in NCCs at early embryonic stage. (*A*) Whole LacZ staining and histological LacZ staining for medial nasal process (MNP) at E10.5 (*n* = 6). Counterstaining for nuclei was performed with nuclear fast red staining. (*B*) Activation of BMP‐SMAD signaling in MNP and lateral nasal process (LNP) was analyzed by phospho‐SMAD 1/5/9 staining (*n* = 6). (*C*) Cell death in MNP was revealed by TUNEL staining (*n* = 6). (*D*, *E*) Number of positive cells for p‐SMAD1/5/9 or TUNEL in each transgenic mouse was quantified. One‐way ANOVA with Tukey's test was used for statistical analysis. NS, no significance; * *p* < 0.05, ** *p* < 0.01. Scale bars: 100 μm.

To address the remaining question of why the region‐specific premature suture fusion patterns developed even though all cranial NCCs at the craniofacial region were similarly recombined, we then hypothesized that cranial NCCs in the facial area at the early embryonic stage might behave differently by BMP signaling between *Wnt1‐Cre* and *P0‐Cre* mice. The origin of cranial NCCs in the cranial sutures remains unclear; however, some reports have shown that cranial NCCs at the first branchial arch and the MNP migrate above the developing eye, then contribute to suture development.^(^ ^45^, ^46^ ^)^ We thus focused on the MNP at an early embryonic stage. Most cranial NCCs in the MNP in *Wnt1‐Cre;LacZ* and *P0‐Cre;LacZ* mice were positive for LacZ (Fig. 7*A*). BMP‐SMAD signaling was significantly increased in the MNP of *Wnt1‐cre;caBmpr1a* and *P0‐Cre;caBmpr1a* mice at E10.5 than the MNP of control mice (Fig. 7*B*,*D*). We have reported that the augmentation of BMP signaling in cranial NCCs caused enhanced cell death.^(^ ^47^ ^)^ Cell death detected with TUNEL staining was significantly increased in *Wnt1‐Cre;caBmpr1a* and *P0‐Cre;caBmpr1a* mice at E10.5 compared with controls; furthermore, *P0‐Cre;caBmpr1a* mice showed more cell death than *Wnt1‐Cre;caBmpr1a* mice (Fig. 7*C*,*E*). It is interesting that BMP‐SMAD signaling levels were similarly upregulated in both mutant mice, while impacts on cell death were different (Fig. 7*D*,*E*). These results suggest that the difference we reported in Cre expressions during the emergence of cranial NCCs (E8‐E9)^(^ ^25^ ^)^ may impact cell survivability at E10.5, which will subsequently affect the behavior of NCC‐derived cells at perinatal stages to differentially contribute suture fusions.

## Discussion

Excessive osteogenic differentiation of suture mesenchymal cells has long been considered the reason for premature suture fusion resulting in craniosynostosis. However, some reports have demonstrated the presence of ectopic cartilage in sutures before their pathologic premature fusion.^(^ ^35^, ^48^ ^)^ Here we demonstrated that cranial NCCs with enhanced BMP signaling developed ectopic cartilage in sutures preceding premature fusion. Our two transgenic mice lines, *Wnt1‐Cre;caBmpr1a* and *P0‐Cre;caBmpr1a*, showed common and specific premature suture fusion patterns, which coincide with the patterns of ectopic cartilage formation found in each mouse line. The ectopic cartilage is replaced by bone nodules, suggesting that the ectopic cartilage prompts endochondral ossification, which is one of the mechanisms of premature suture fusion. Our histological observations at early embryonic stages, together with the fact that NCSCs isolated from *P0‐Cre;caBmpr1a* mice showed robust chondrogenic ability, imply that augmentation of BMP signaling at early embryonic stages alters the behaviors of cranial NCCs toward chondrogenic differentiation. BMPs are known as bone inducers^(^ ^49^, ^50^ ^)^; we found hypomorphic calvarial bone formation in both *Wnt1‐Cre;caBmpr1a* and *P0‐Cre;caBmpr1a* mice. Previously, we reported that augmented BMP signaling in cranial neural crest cells resulted in p53‐dependent cell death^(^ ^47^ ^)^ and that in osteoblasts it does not affect bone mass.^(^ ^51^ ^)^ Together with new findings in this report, we speculate that BMP signaling in neural crest progenitors positively affects their commitment to chondrogenic differentiation and BMP signaling in cells committed to the osteogenic lineage does not affect their bone‐forming activity but rather prompts p53‐dependent cell death, leading to hypomorphic bone formation in the mutant mice we investigated in this report.

### Endochondral ossification in cranial suture mesenchyme is a potential reason for premature suture fusion

Cranial NCCs are multipotent cells differentiating into osteoblasts, chondrocytes, and others^(^ ^52^ ^)^; however, cranial NCCs in calvaria do not differentiate into chondrocytes under physiological condition. The PF suture fuses in normal development, followed by cartilage formation in the area of the suture.^(^ ^31^ ^)^ We recently reported that augmentation of BMP signaling in cranial NCCs alters cell fate toward the chondrogenic lineage at early embryonic stages.^(^ ^41^ ^)^ In the present report, we showed a significant increase in the cell death of cranial NCCs from *P0‐Cre;caBmpr1a* mice compared with *Wnt1‐Cre;caBmpr1a* mice in the facial primordia (Fig. 7*C*,*E*) at much earlier stages than perinatal stages when ectopic cartilage formed (Fig. 3). This indicates that the changes in the behavior of cranial NCCs at early embryonic stages may influence chondrogenic differentiation at later stages. Such large temporal differences support the idea that a mechanism of ectopic cartilage formation may be due to cell fate switching toward chondrogenic lineage rather than prompting chondrogenic differentiation of committed progenitors.

### *Wnt1‐Cre* and *P0‐Cre* label different subpopulations of cranial NCCs

Cranial NCCs develop the face and anterior part of the head.^(^ ^4^ ^)^ We have shown that *Wnt1‐Cre* and *P0‐Cre* markers overlap but with unique domains of NCCs at the stage of NCC formation (E8‐E9); more *Wnt1‐Cre* expressing cells are found in the midbrain region while more *P0‐Cre* expressing cells are found in the hindbrain region.^(^ ^25^ ^)^ Moreover, our study also demonstrated that *Wnt1‐Cre* expressing cells appear at five somite stages and *P0‐Cre* expressing cells appear at 11 somite stages,^(^ ^25^ ^)^ suggesting that *Wnt1‐Cre* expressing cells and *P0‐Cre* expressing cells appear at different places and with different timings. It is known that NCCs have subpopulations, mainly cranial NCCs, cardiac NCCs, and trunk NCCs, and they are assigned by where they emerge.^(^ ^52^ ^)^ Also, their differentiation potentials are different. It has been reported that cranial NCCs at the forebrain mainly give rise to frontal and nasal bone, including AF suture, while those at the midbrain mainly give rise to maxillary and dentary bones, including nasal‐maxillary sutures.^(^ ^52^ ^)^ Therefore, it is possible that cranial NCCs from different positions, depicted by *Wnt1‐Cre* and *P0‐Cre* recombination patterns, have a different character over time, resulting in premature suture fusion in different places through a mosaic activation pattern of BMP signaling.

In summary, we propose that the augmentation of BMP signaling develops ectopic cartilage prompting ectopic endochondral ossification in the calvaria, ultimately resulting in premature suture fusion and a craniosynostosis phenotype. It is still unclear whether inhibition of ectopic endochondral ossification in the sutures can rescue premature suture fusion. It is an important future effort to identify the critical time window that enhanced BMP signaling in cranial NCCs influences cell fate specification. Although there are some gaps between the translation from findings in mouse models into clinical research, we hope these findings will contribute to defining the molecular cellular mechanisms that might contribute to the etiology of craniosynostosis, eventually leading to better treatment options for pathological conditions.

## Author Contributions

**Hiroki Ueharu:** Data curation; formal analysis; investigation; methodology; project administration; validation; visualization; writing – original draft. **Haichun Pan:** Data curation; formal analysis; investigation; methodology; validation; writing – review and editing. **Xia Liu:** Data curation; investigation; methodology; writing – review and editing. **Mamoru Ishii:** Methodology; writing – review and editing. **Jessica Pongetti:** Data curation; formal analysis; methodology; writing – review and editing. **Anshul Kulkarni:** Formal analysis; methodology; writing – review and editing. **Folasade Adegbenro:** Formal analysis; methodology; writing – review and editing. **Jaden Wurn:** Data curation; formal analysis; methodology; writing – review and editing. **Robert E. Maxson:** Methodology; writing – review and editing. **Hongchen Sun:** Investigation; methodology; writing – review and editing. **Yoshihiro Komatsu:** Investigation; methodology; writing – review and editing. **Honghao Zhang:** Data curation; formal analysis; investigation; supervision; writing – original draft; writing – review and editing. **Jingwen Yang:** Data curation; formal analysis; methodology; project administration; writing – original draft; writing – review and editing. **Yuji Mishina:** Conceptualization; funding acquisition; investigation; project administration; supervision; writing – original draft; writing – review and editing.

## Funding Source

This work was supported by grants from the NIH (R01DE020843 and R01DE027662 to YM). The molecular biology core at the School of Dentistry is funded by NIH/P30AR069620. The μCT core at the University of Michigan School of Dentistry is funded in part by NIH/NCRR S10RR026475‐01. HU was supported, in part, by the Uehara Memorial Foundation postdoctoral fellowship in Japan.

## Conflict of Interest

All authors state that they have no conflict of interest. The authors declared no potential conflicts of interest with respect to the research, authorship, and/or publication of this article.

### Peer Review

The peer review history for this article is available at https://publons.com/publon/10.1002/jbm4.10716.

## Supporting information


**Figure S1.** Positions of landmarks listed in Table S1 and S2.

**Table S1.** List of landmarks for linear distance analysis.
**Table S2.** List of linear distance analysis.
**Table S3.** Linear distances between landmarks indicate abnormal skull shape in *P0‐Cre;caBmpr1a* mice. Length of each mouse is shown in millimeters. Measurements with statistical significances are highlighted in yellow. Control, *n* = 15, mutant, *n* = 10. (Excel file)
**Table S4.** Linear distances between landmarks indicate abnormal skull shape in *Wnt1‐Cre;caBmpr1a* mice. Length of each mouse is shown in millimeters. Measurements with statistical significances or tendency are highlighted in yellow or green. Control, *n* = 4, mutant, *n* = 5. (Excel file)
**Table S5.** Primers for qRT‐PCR
**Table S6.** Isolation of neural crest stem cells

## Data Availability

Data available on request from the authors.
