# Supplementary figures and images for: Augmentation of BMP Signaling in Cranial Neural Crest Cells Leads to Premature Cranial Sutures Fusion through Endochondral Ossification in Mice
Source: JBMR Plus. 2023 Feb 23;7(4):e10716. doi: 10.1002/jbm4.10716 (PMC10097634; doi:10.1002/jbm4.10716)

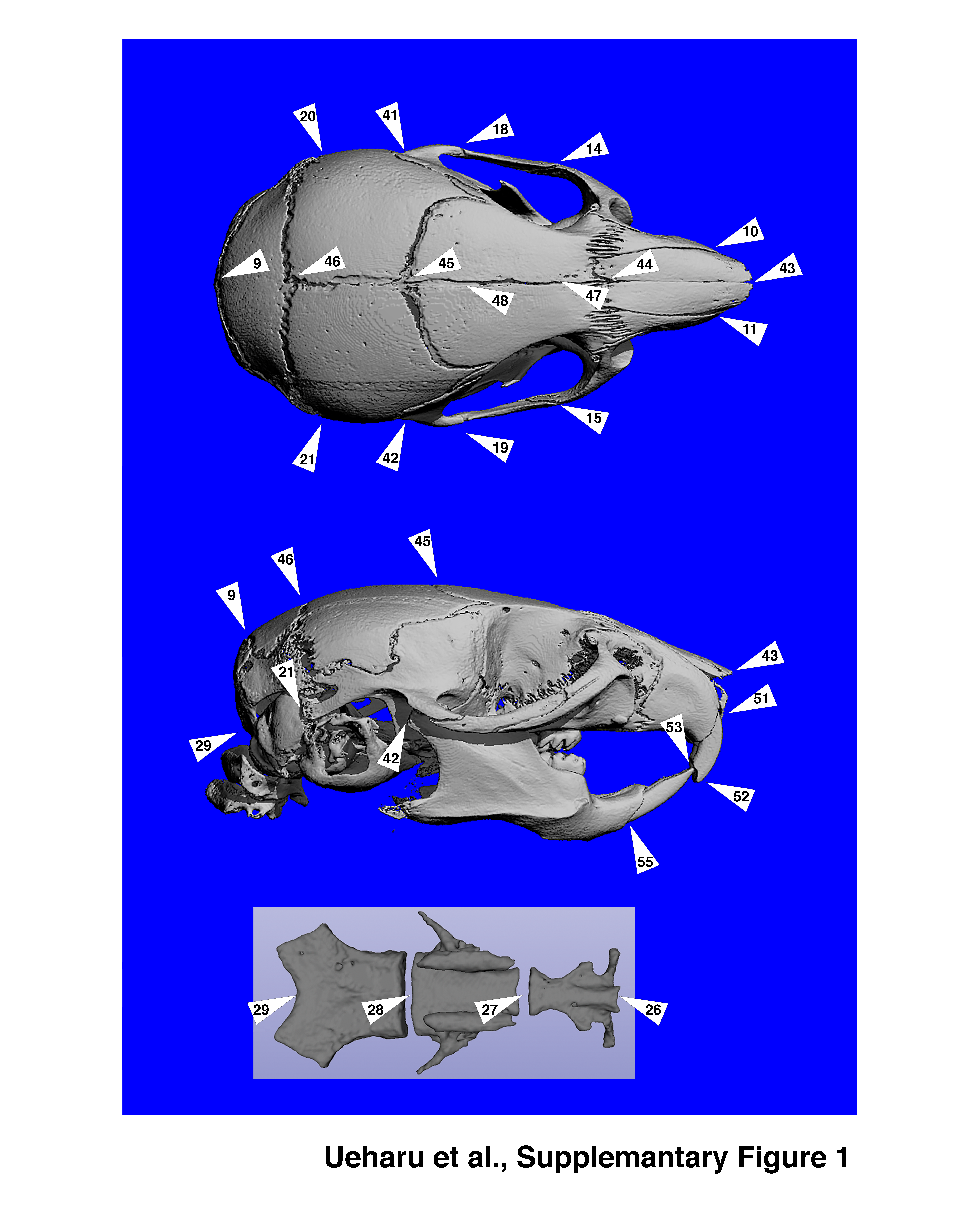

Supplement: Supplementary file 1 — Figure S1. Positions of landmarks listed in Table S1 and S2. [file JBM4-7-e10716-s002.tif]
