# Supplementary material for: Augmentation of BMP Signaling in Cranial Neural Crest Cells Leads to Premature Cranial Sutures Fusion through Endochondral Ossification in Mice
Source: JBMR Plus. 2023 Feb 23;7(4):e10716. doi: 10.1002/jbm4.10716 (PMC10097634; doi:10.1002/jbm4.10716)
Supplement: Supplementary file 2 — Table S1. List of landmarks for linear distance analysis. Table S2. List of linear distance analysis. Table S3. Linear distances between landmarks indicate abnormal skull shape in P0‐Cre;caBmpr1a mice. Length of each mouse is shown in millimeters. Measurements with statistical significances are highlighted in yellow. Control, n = 15, mutant, n = 10. (Excel file) Table S4. Linear distances between landmarks indicate abnormal skull shape in Wnt1‐Cre;caBmpr1a mice. Length of each mouse is shown in millimeters. Measurements with statistical significances or tendency are highlighted in yellow or green. Control, n = 4, mutant, n = 5. (Excel file) Table S5. Primers for qRT‐PCR Table S6. Isolation of neural crest stem cells [file JBM4-7-e10716-s001.zip › jbm410716-sup-0002-TablesS1-S6/jbm410716-sup-0002-Tables.docx]

**Table S1. List of landmarks for linear distances analysis.**

| Anatomical landmarks | Description |
| --- | --- |
| 1,2 (43) | A, Nasale, Rostral point of nasal bone |
| 3,4 (44) | N, Nasion, Caudal point of nasal bone |
| 5,6 (45) | F, Caudal point of frontal bone |
| 7,8 (46) | Pr, Caudal point of Parietal bone |
| 9 | Pri, Most posterior point of interparietal bone at midline |
| 10,11 | XN, Most anterior point at intersection of premaxillae and nasal bones |
| 12,13 | XMx, Most inferior point of premaxilla at Premaxilla-maxilla suture |
| 14,15 (47) | ZMx, Most anterior point of zygomatic process of zygoma at intersection of maxilla and zygoma |
| 16,17 | MxSp, Most posterior point of alveolar ridge at the Intersection of maxilla and sphenoid |
| 18,19 (48) | ZT, Most anterior point of Zygomatic process of temporal bone at intersection of zygoma and zygomatic process of temporal bone |
| 20,21 | Tp, Most posterior portion of post-tympanic hook of temporal bone |
| 22,23 (49) | PNS, posterior nasal spine |
| 24,25 (50) | Pf, Posterior palatine fissure |
| 26 | Sp, Intersection of presphenoid and frontal bone at midline of cranial base |
| 27 | Iss, at ISS, intersphenoidal suture, most anterosuperior point |
| 28 | Sos, at SOS, sphenoid-occipital suture, most anterosuperior point |
| 29 | Ba, Basion |
| 30 | Op, Opisthion |
| 31,32 (51) | MxAl, Most inferior point at center of alveolar ridge on the lingual side of maxillary incisor, lingual |
| 33,34 (52) | MxC, Maxillary incisor edge |
| 35,36 (53) | Mandibular incisal edge |
| 37,38 (54) | Deepest part of the antegonial notch curvature |
| 39,40 (55) | Most anterior point at center of alveolar ridge on the lingual side of mandibular incisor, lingual |
| 41,42 (56) | Most posterior point of condyle |

**Table S2. List of linear distances analysis.**

| Linear measurements | Anatomical landmarks |
| --- | --- |
| Total skull length | 43-9 |
| **Nasomaxillary complex** | |
| Nasal bone length | 43-44 |
| Frontal bone length | 44-45 |
| Width of nasal bone at intersection with premaxillae | 10-11 |
| Zygomatic arch length | 47-48 |
| Erupted upper incisor length | 51-52 |
| Viscerocranial length | 45-51 |
| **Calvaria** | |
| Parietal length | 45-46 |
| Interparietal length | 46-9 |
| Occipital Bone | 9-30 |
| Width at Zygomatic arch (anterior) | 14-15 |
| Width at Zygomatic arch (posterior) | 18-19 |
| Width of temporal bone | 20-21 |
| **Cranial Base** | |
| Total Cranial Base | 43-29 |
| Cranial base length I | 26-28 |
| Cranial base length II | 26-29 |
| Presphenoid length | 26-27 |
| Basiosphenoid length | 27-28 |
| Basioccipital length | 28-29 |
| **Viscerocranial heights** | |
| Viscerocranial height at ISS | 45-27 |
| Viscerocranial height at SOS | 45-28 |
| Viscerocranial height at basion | 45-29 |

**Table S5. Primers for QRT-PCR**

----------------------------------------------------------------------------------------------------------------

Gene Forward Reverse

----------------------------------------------------------------------------------------------------------------

β-actin 5’-CGCATCCTCTTCCTCCCTGG-3’ 5’-GTGGTACCACCAGACAGCAC-3’

Snail1 5’-CTTGTGTCTGCACGACCTGT-3’ 5’-CTTCACATCCGAGTGGGTTT-3’

Twist1 5’-GGAGGATGGAGGGGGCCTGG-3’ 5’-TGTGCCCCACGCCCTGATTC-3’

Sca-1 5’-CTCTGAGGATGGACACTTCT-3’ 5’-GGTCTGCAGGAGGACTGAGC-3’

CD44 5’-GTGGCACACAGCTTGGGGA-3’ 5’-TCAGAGCCAGTGCCAGGAGAGAT-3’

Nestin 5’-AATGGGAGGATGGAGAATGGAC-3’ 5’-TAGACAGGCAGGGCTAGCAAG-3’

----------------------------------------------------------------------------------------------------------------

**Table S6. Isolation of neural crest stem cells**

----------------------------------------------------------------------------------------------------------------

controls *P0-Cre;caBmpr1a*

----------------------------------------------------------------------------------------------------------------

Total litters 8 8

Number of embryos started with 22 24

Number of embryos FACS isolated 12 15

Number of clones cultured over passage 10 8 8

Number of clones cultured over passage 20 8 8

----------------------------------------------------------------------------------------------------------------
